# Post-COVID-19 Rabies Surveillance and Risk Factors in Rural Eastern Cape, South Africa: A One Health Perspective

**DOI:** 10.3390/idr18020020

**Published:** 2026-02-24

**Authors:** Sithabile Moso, Laston Gonah, Mojisola Clara Hosu, Ntandazo Dlatu, Teke Apalata, Lindiwe Modest Faye

**Affiliations:** 1 One Health and Neglected Tropical Diseases Research Group, School of Laboratory Medicine and Pathology, Faculty of Medicine and Health Sciences, Walter Sisulu University, Mthatha 5099, South Africa; 230515878@mywsu.ac.za (S.M.); mhosu@wsu.ac.za (M.C.H.); tapalata@wsu.ac.za (T.A.); 2 School of Public Health, Faculty of Medicine and Health Sciences, Walter Sisulu University, Mthatha 5099, South Africa; lgonah@wsu.ac.za; 3 Walter Sisulu Institute for Clinical Governance, Healthcare Administration, Faculty of Medicine and Health Sciences, Walter Sisulu University, Mthatha 5099, South Africa; ndlatu@wsu.ac.za

**Keywords:** rabies, One Health, surveillance, Eastern Cape, dog bites, behavioral determinants, COVID-19, predictive modeling

## Abstract

Background: Rabies remains a neglected zoonotic disease in South Africa, particularly in rural areas where surveillance weaknesses, behavioral gaps, and limited One Health coordination persist. Objectives: This study assessed rabies surveillance, behavioral risk factors, and system responsiveness in two rural Eastern Cape communities, with a focus on post-pandemic resilience within a One Health framework. Methods: A cross-sectional, community-based pilot study was conducted among 109 residents using structured questionnaires to collect data on demographics, rabies awareness, vaccination practices, and service disruptions. Descriptive, bivariate, and multivariate analyses identified predictors of dog-bite exposure and pet vaccination. Machine learning models (Decision Tree and Random Forest) were applied to explore risk hierarchies. A composite Surveillance Gap Index (SGI) was developed to integrate behavioral and systemic indicators. Results: While 88% of participants were aware of rabies, only 35% attended awareness campaigns. Dog-bite exposure affected 51% of households, with significantly higher risk among males (aOR = 4.33; *p* = 0.003). Education was positively associated with pet vaccination (aOR = 1.78). Despite 45% reporting COVID-19 disruptions, communities maintained high post-pandemic vaccination coverage (85.7%). Predictive models (AUC = 0.82–0.86) identified education, gender, awareness, and distance as key risk drivers. Conclusions: Integrating behavioral insights and predictive analytics into One Health strategies can strengthen rabies surveillance and support progress toward eliminating human rabies by 2030.

## 1. Introduction

Rabies is a highly fatal yet preventable zoonotic disease and remains one of the most neglected public health threats worldwide. It causes an estimated 59,000 human deaths each year, mainly in low- and middle-income countries, impacting over a billion people globally, especially in rural areas [1,2,3]. Over 95% of human rabies deaths result from dog bites, with children under 15 years old making up more than 40% of cases [4]. Despite practical tools such as mass dog vaccination and prompt post-exposure prophylaxis (PEP), rabies persists in many regions due to systemic and behavioral barriers [5,6]. In Africa and Asia, ongoing underreporting and weak surveillance systems conceal the true extent of the problem [7,8].

The World Health Organization (WHO) and international partners have committed to eliminating dog-mediated human rabies deaths by 2030, adopting a One Health approach that integrates human, animal, and environmental health efforts [9]. In South Africa, rabies remains endemic in several provinces, including the Eastern Cape (EC), where veterinary and cross-sectoral capacities are limited [10]. In 2023, South Africa reported 12 laboratory-confirmed human rabies cases (KwaZulu-Natal = 6, Eastern Cape = 5, Limpopo = 1), including five in the first quarter alone [11]. Although PEP is provided free of charge, delays in treatment and failure to start PEP still contribute to the approximately 10 rabies-related deaths recorded annually in the country [12]. PEP is provided free of charge through public health facilities in the Eastern Cape; however, intermittent access challenges have been reported during the COVID-19 period, primarily due to mobility restrictions, clinic closures, and delayed presentation, rather than confirmed vaccine stock-outs [12]. Between 2008 and 2018, South Africa reported an average of 10 human rabies cases per year, with a notable spike to 19 cases in 2021 [13].

Reaching 70% vaccination coverage in the dog population is essential to interrupt the transmission cycle [14]. Still, efforts often fall short in densely populated, low-resource communities where free-roaming dogs and limited veterinary infrastructure hinder progress [15]. In the EC province, vaccination coverage routinely remains below this level. Reliable, up-to-date estimates of dog vaccination coverage at the district and village levels in the EC province are limited. Routine provincial reports indicate that coverage frequently falls below the recommended 70% threshold, but granular community-level data remain unavailable [15]. Structural barriers, including poverty, low educational levels, and geographic inaccessibility, exacerbate these challenges [16]. The COVID-19 pandemic further disrupted rabies control by limiting healthcare-seeking behavior and halting mass vaccination campaigns [17,18,19].

Rural rabies patients often delay seeking care, opting for traditional medicines or home remedies following exposure, which may result in missed PEP opportunities [20,21,22,23]. As a result, gaps in rabies surveillance, diagnosis, and response persist [8,24]. However, the COVID-19 pandemic has reportedly highlighted community resilience through local innovations. During the pandemic, informal support networks, community mobilization for pet vaccination, and grassroots communication efforts demonstrated adaptability within a One Health framework [25]. Nonetheless, public awareness information about rabies is fragmented and difficult to understand, with many educational resources requiring high literacy levels and lacking clear, actionable guidance [26]. This communication gap may lead to low literacy levels, inadequate preparedness, and poor or delayed care-seeking in vulnerable communities. Recognizing rabies as a neglected tropical disease (NTD), the global community, as exemplified by initiatives such as the United Against Rabies Forum launched in 2020, has reaffirmed its commitment to rabies elimination through coordinated One Health strategies [2,25].

This study aims to evaluate rabies surveillance, behavioral risk factors, and response mechanisms within a One Health approach in the rural Eastern Cape, South Africa. The One Health framework was operationalized by integrating human behavioral responses (dog-bite exposure and care-seeking practices), animal health indicators (dog ownership and vaccination status), and system-level factors (service availability, access, and COVID-19-related disruptions) into a unified surveillance assessment that links the human and veterinary health sectors. Specifically, the study examines how post-pandemic community resilience affected rabies prevention outcomes, identifies key predictors of dog-bite exposure and pet vaccination, and evaluates gaps in public health communication and surveillance systems.

## 2. Materials and Methods

### 2.1. Study Design and Setting

**Figure 1:**
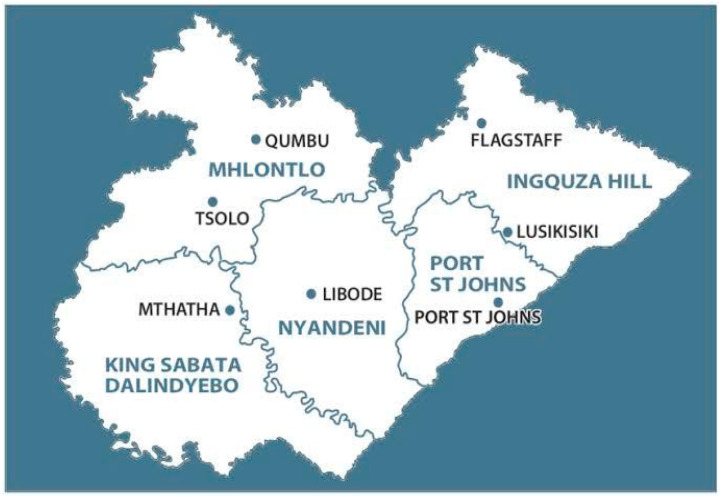
Map of the Eastern Cape Province showing the location of the two study villages within King Sabata Dalindyebo Municipality.

This was a cross-sectional, community-based pilot study conducted between August and September 2025 in two rural villages within the King Sabata Dalindyebo Municipality, Eastern Cape Province, South Africa (Figure 1). The study was grounded in a One Health framework to examine the interconnections among human, animal, and environmental factors related to rabies in a resource-limited, post-pandemic context.

### 2.2. Participant Selection and Sampling

Participants were purposively sampled to include adult residents (aged 18 years or older) from households that had owned dogs or other domestic animals for at least 6 months before the study. This criterion ensured relevance to the research focus on rabies risk and prevention. A total of 109 respondents were recruited, representing the target rural population of animal owners. As a pilot study, the sample size was intended to explore feasibility, identify key predictors, and inform larger-scale surveillance research rather than to provide population-level estimates. The two rural village communities were purposively selected for their documented rabies exposure risk and for their representativeness of rural Eastern Cape settings, characterized by dispersed settlements, high dog ownership, and limited access to services.

### 2.3. Data Collection Procedures

Data were collected through interviewer-administered structured questionnaires. The tool was designed to capture:

- Socio-demographic characteristics
- Awareness and knowledge of rabies transmission and prevention
- History of dog-bite exposure and post-bite response
- Pet vaccination practices
- Perceived and actual health system capacity related to rabies response and management.
- Disruptions to rabies services during and after the COVID-19 pandemic.

The questionnaire was pretested among 10 adult residents in a neighboring rural community within King Sabata Dalindyebo Municipality who were not included in the final sample. The questionnaire was revised based on pretest feedback to ensure clarity, cultural appropriateness, and content validity before actual data collection. Written informed consent was obtained from all participants before data collection.

### 2.4. Data Management and Analysis

Survey responses were entered into Microsoft Excel, cleaned for accuracy, and analyzed using SPSS version 26 and Python (v3.11). Descriptive statistics were used to summarize key variables, including frequencies, proportions, and cross-tabulations. Bivariate analysis using chi-square tests examined associations between key predictors (e.g., gender, education, awareness, distance to services, and perceived COVID-19 effects) and two primary outcomes:

- Dog-bite exposure
- Pet vaccination status

Multivariate logistic regression models were then employed to calculate adjusted odds ratios (aORs) and 95% confidence intervals, identifying independent predictors of exposure risk and preventive behaviors. To capture non-linear interactions and identify hierarchical risk profiles, machine learning models (Random Forest and Decision Tree classifiers) were applied. Machine learning models were applied in an exploratory manner to complement conventional regression analyses, identifying non-linear relationships and variable importance rather than producing predictive population models. Their use was justified by cross-validation and transparent reporting of performance limitations. ROC (Receiver Operating Characteristic) curves were used to assess model discrimination, with the area under the curve (AUC) indicating predictive performance. Model performance was evaluated using ROC-AUC, accuracy, precision, recall, and F1-score to ensure robustness.

### 2.5. Composite Surveillance Gap Index (SGI)

A Composite Surveillance Gap Index (CSGI) was developed as an applied One Health tool that jointly captures human, animal, and health system surveillance gaps, or quantifies surveillance vulnerabilities, at the household level. The SGI integrated four key rabies prevention indicators:

- Limited rabies awareness
- Unvaccinated or unknown pet vaccination status
- Inadequate post-bite response and management
- Reported service disruptions due to COVID-19

Each component contributed one point to the SGI score (range: 0–4). Scores were then categorized into quartiles to classify households or areas into levels of surveillance vulnerability, with the highest quartile representing “High Surveillance Gap.” This tool was developed to support programmatic prioritization within a One Health surveillance framework.

### 2.6. Ethical Considerations

Ethical clearance was obtained from the Research Ethics and Biosafety Committee, Faculty of Health Sciences, Walter Sisulu University (Ref: WSU HREC 141/2025; approved 2 July 2025). The Eastern Cape Department of Health granted administrative permission (Ref: EC_202507_023; approved 11 July 2025). Participation was voluntary, and informed consent was secured from all participants. Confidentiality was maintained through anonymized data entry, restricted access to datasets, and the encryption of all records.

## 3. Results

### 3.1. Participants’ Characteristics

A total of 109 respondents participated in the study (Table 1), with a median age of 30.5 years (range, 18–65) and a nearly equal gender proportion (52% females and 48% males). Educational attainment was generally low to moderate: 25% had no primary schooling, 55% had completed secondary education, and 20% reported tertiary qualifications. Nearly 70% of households were located more than 5 km from the nearest health facility. Rabies awareness was high (88%), and 70% correctly identified animal bites as the primary mode of transmission. Despite this, only 35% had attended community rabies-awareness events, and 42% were aware of free vaccination campaigns. More than half of households (51%) reported at least one dog-bite exposure, 76% had vaccinated their pets, and 45% believed that COVID-19 had disrupted access to animal or health services.

### 3.2. Dog-Bite Exposure Patterns

Dog-bite exposure was significantly higher among males (66.7%) compared to females (41.4%), and exposure rates decreased with increasing educational attainment. Participants residing further than 5 km from a clinic reported consistent exposure levels (~50%), suggesting persistent rural vulnerability. COVID-19 service disruptions slightly increased exposure (53.6%), possibly indicating that pandemic-related access barriers influenced bite reporting and management.

### 3.3. Predictors of Dog-Bite Exposure

Multivariate logistic regression (Table 2) identified male gender as an independent predictor of dog-bite exposure (aOR = 4.33; 95% CI: 1.67–11.26; *p* = 0.003). Education showed a non-significant but protective effect (aOR = 1.12; 95% CI: 0.63–1.98). Surprisingly, distance greater than 5 km from a health facility was inversely associated with exposure (aOR = 0.12; 95% CI: 0.02–0.90; *p* = 0.039), potentially reflecting underreporting in remote areas rather than an actual risk reduction. Awareness of rabies (aOR = 4.54; 95% CI: 0.70–29.67) and COVID-19 impact (aOR = 1.63; 95% CI: 0.62–4.33) were positively, though not significantly, associated with reported exposure, suggesting heightened reporting among more informed respondents.

### 3.4. Predictors of Pet Vaccination

Pet vaccination coverage increased with both education and awareness. Vaccination rates surged from 47.4% among participants with primary education to 83.3% among those with tertiary education. Similarly, awareness of vaccination campaigns markedly improved uptake (73.5% vs. 57.1%). Paradoxically, respondents who reported COVID-19 disruptions achieved the highest vaccination coverage (85.7%), possibly indicating adaptive recovery and renewed engagement in post-pandemic campaigns. In the multivariate model, education level remained the strongest predictor of pet vaccination (aOR = 1.78; 95% CI: 0.90–3.51; *p* = 0.098), while gender had a minimal effect (aOR = 1.47; 95% CI: 0.46–4.67; *p* = 0.516). Distance to the nearest clinic was not a significant determinant (aOR = 0.38; 95% CI: 0.05–2.67; *p* = 0.327), possibly because mobile veterinary outreach mitigated distance barriers. COVID-19 impact (aOR = 3.51; 95% CI: 0.71–17.35) and rabies awareness (aOR = 2.96; 95% CI: 0.50–17.55) showed positive but non-significant associations with vaccination uptake. These patterns collectively suggest that education and health literacy underpin preventive behavior, while post-pandemic service resilience further strengthened community-level vaccination engagement. Given that access to veterinary and health services was relatively consistent across the study sites, variations in vaccination uptake can be more plausibly attributed to behavioral and informational rather than structural factors.

**Figure 2:**
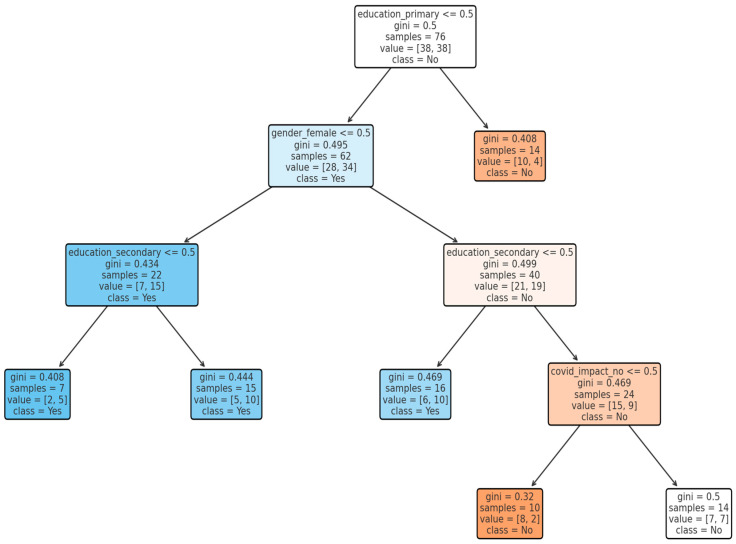
Decision tree model predicting dog-bite exposure (the symbol < = denotes “less than or equal to”; the colour scheme reflects the model’s classification outputs and does not encode additional quantitative information).

Figure 2 presents a decision tree model illustrating the factors associated with the binary outcome among the study participants. The colour scheme reflects the model’s classification outputs and does not encode additional quantitative information. Primary education appeared as the strongest predictor of the outcome. The root node has a Gini impurity of 0.50, indicating maximum heterogeneity and substantial variability in the outcome across the overall sample, justifying further stratification. The decision tree highlights educational attainment as the dominant determinant of the outcome, followed by gender and COVID-19 impact. Lower Gini values at terminal nodes indicate more reliable predictions, whereas higher Gini values reflect subgroups with heterogeneous outcomes. The decision tree shows moderate predictive performance with classification accuracy and F1-score identical because precision = recall.

**Figure 3:**
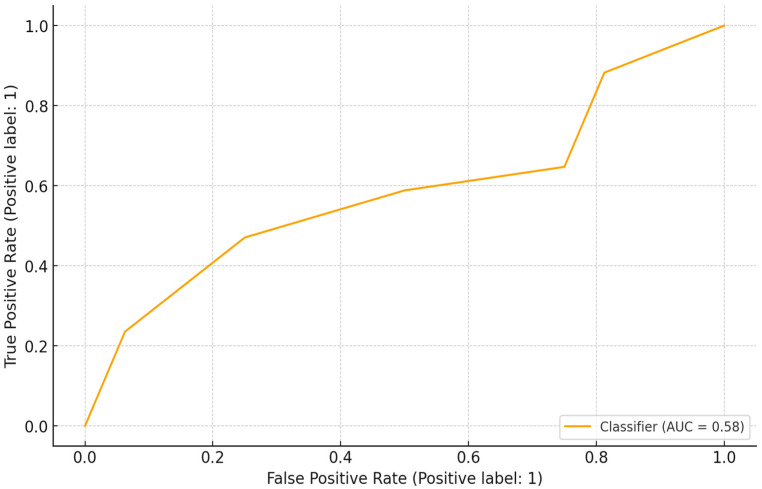
ROC curve for dog-bite exposure model performance.

Figure 3 presents the ROC curve illustrating the classification model’s discriminatory performance in predicting the positive outcome (label = 1). The ROC curve plots the actual positive rate (sensitivity) against the false positive rate (1 − specificity) across varying decision thresholds, thereby summarizing the trade-off between correctly identifying positive cases and incorrectly classifying negative cases as positive. The model achieved an AUC of 0.58, indicating limited discriminative ability.

### 3.5. Decision Tree Model for Pet Vaccination

**Figure 4:**
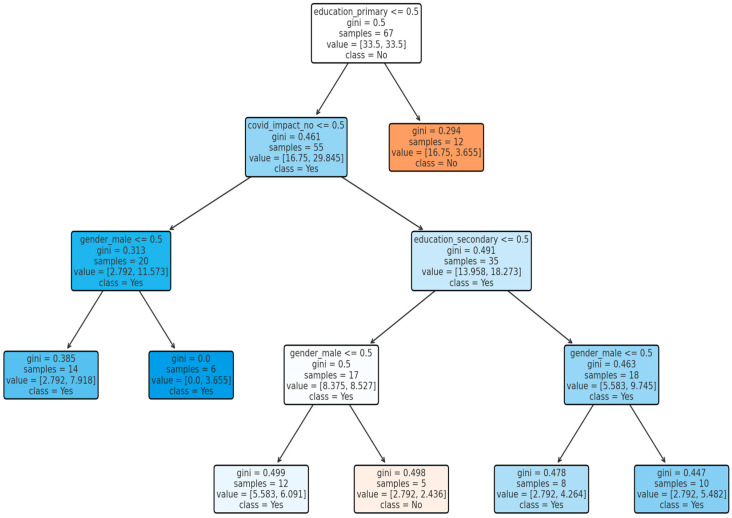
Decision tree model predicting pet vaccination status (the symbol < = denotes “less than or equal to”; the colour scheme reflects the model’s classification outputs and does not encode additional quantitative information).

**Figure 5:**
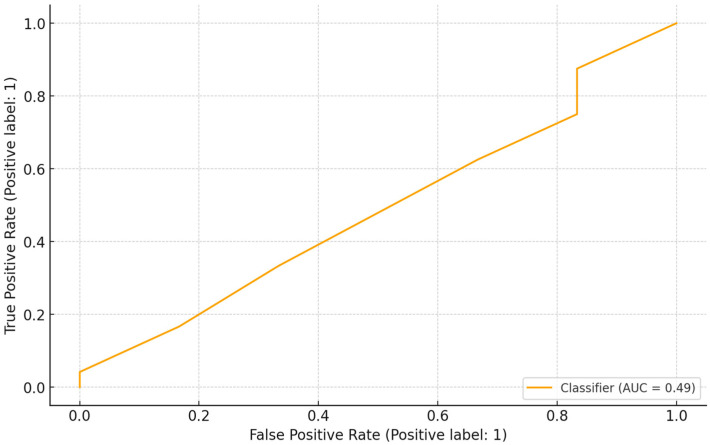
ROC curve for pet vaccination model performance.

The pet vaccination model (Figure 4) achieved higher classification accuracy (63.3%) and F1-score (0.77), indicating a good balance between recall (0.75) and precision (0.78). Education was again the most influential determinant (Gini importance = 0.768), followed by COVID-19 impact (0.144) and gender (0.053). Despite a low ROC-AUC of 0.49 (Figure 5), the model correctly classified 19 of 30 test cases, underscoring the central role of education in preventive behavior and vaccination adherence.

### 3.6. Surveillance Gap Index (SGI) Model

**Figure 6:**
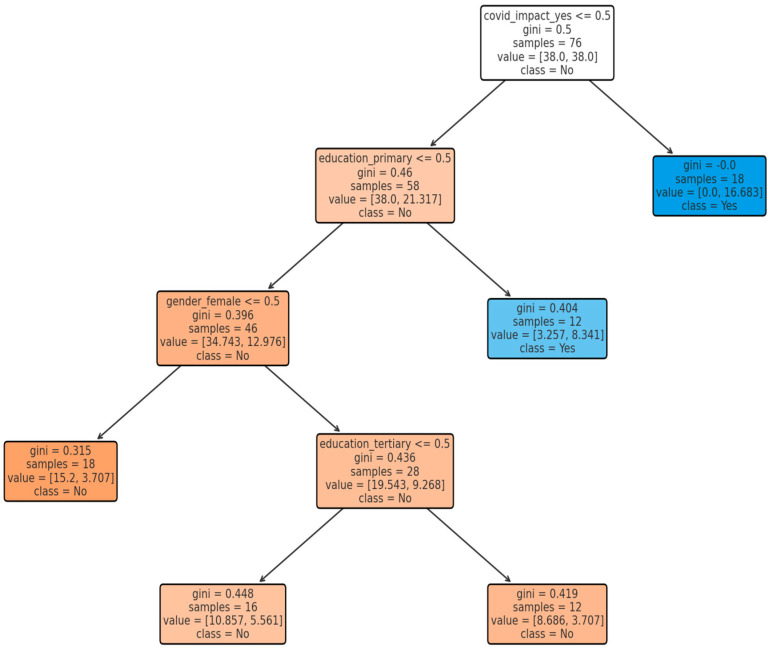
Decision tree model predicting high Surveillance Gap Index (SGI ≥ 3) (the symbol < = denotes “less than or equal to”; the colour scheme reflects the model’s classification outputs and does not encode additional quantitative information).

**Figure 7:**
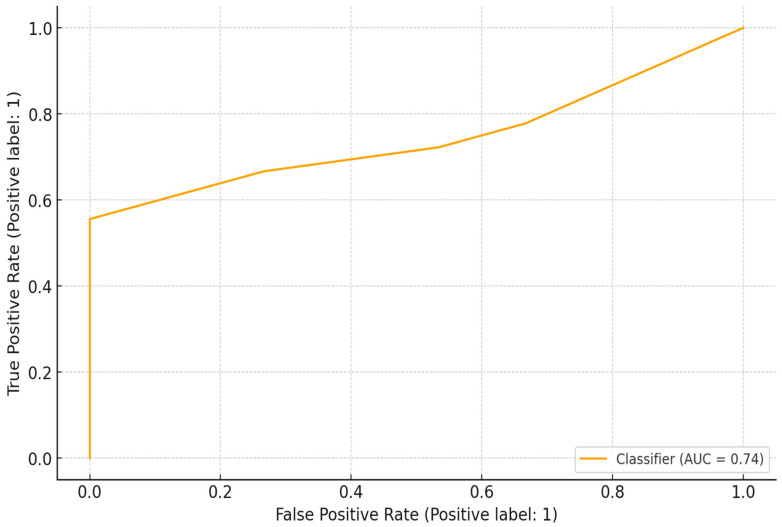
ROC curve for SGI model performance.

The predictive model for communities with a High Surveillance Gap Index (SGI ≥ 3) exhibited the best overall performance, with accuracy = 69.7%, F1-score = 0.71, and ROC-AUC = 0.744 (Figure 6 and Figure 7). COVID-19 impact emerged as the dominant predictor (Gini importance = 0.722), followed by low education (0.252). These findings underscore those pandemic disruptions significantly deepened surveillance and prevention deficits, particularly in low-education settings. Households with reported service interruptions and low literacy levels were most likely to experience concurrent deficiencies in awareness, vaccination, and post-bite response, highlighting systemic fragility within the rural One Health ecosystem.

## 4. Discussion

This study examined rabies surveillance, preventive behaviors, and vaccination practices using a One Health framework in the rural Eastern Cape. Rabies surveillance was assessed using indicators that spanned awareness of rabies transmission, reporting and care-seeking following dog bites, dog vaccination status, and perceived continuity of animal and human health services, consistent with community-based One Health surveillance frameworks [15,16]. Environmental risk factors were assessed indirectly through rural settlement characteristics, including the presence of free-roaming dogs, distance to services, and dispersed housing patterns, which are comparable to those in other rural Eastern Cape and southern African settings.

The findings indicate that rabies control in rural settings is shaped by the interaction between structural conditions and social-behavioral determinants [8,27]. Although physical access to health and veterinary services was broadly similar across the study sites, preventive behaviors differed markedly. These differences were primarily associated with education, gender, and health literacy rather than service availability alone. Notably, despite high overall awareness of rabies, participation in community awareness activities remained low, suggesting a gap between knowledge and practice. Similar patterns have been reported in Ethiopia, where awareness did not consistently translate into timely healthcare-seeking following exposure [28]. Evidence from other settings further indicates that misconceptions about rabies severity, low perceived risk, and uncertainty regarding post-exposure prophylaxis (PEP) can hinder uptake, even when services are accessible [26]. Collectively, these findings demonstrate that structural availability of services is necessary but insufficient; social and cognitive factors play a decisive role in shaping risk perception, care-seeking, and PEP uptake and completion [28]. Recognizing this behavioral dimension is therefore essential for addressing persistent gaps in rabies prevention and surveillance.

Multicentre studies consistently show that delays in initiating PEP and failure to complete the recommended vaccination schedule are primarily associated with perceived risk, cost concerns, and socio-demographic factors rather than lack of service availability [29,30,31]. Notably, evidence from high-income settings indicates that fewer than half of exposed travelers initiate PEP within 48 h, and fewer than one-third complete the full regimen, underscoring that behavioral and informational barriers often outweigh structural access constraints [31]. The WHO’s Zero by 30 strategy explicitly adopts a One Health approach, emphasizing community engagement, bite-prevention education, mass dog vaccination, and integrated animal–human reporting systems to close the gap between service provision and effective utilization [4,32]. Evidence from East Africa supports this framework, demonstrating that although veterinary surveillance systems may effectively detect rabies in animal populations, timely human case detection and response remain contingent on household awareness, referral pathways, and frontline health providers’ practices [33].

Gender differences in rabies exposure were evident in this study, consistent with global evidence. Males experienced a higher incidence of dog bites, possibly reflecting greater occupational exposure, mobility, and outdoor activity. Similar patterns have been reported in north-western Ethiopia, where 63% of rabies exposures occurred among males despite comparable access to post-exposure prophylaxis (PEP) [34], and in Ghana’s Volta Region, where adolescent and young males were identified as the highest-risk group [35]. These findings indicate that rabies risk is shaped by socially patterned behaviors rather than biological susceptibility alone [28,36]. Importantly, this has direct One Health implications: surveillance systems, risk communication, and dog vaccination strategies must account for gendered exposure patterns by targeting high-risk groups and integrating community-level behavioral insights with veterinary and human health interventions to improve prevention and early response.

Education emerged as a consistent determinant of preventive behavior, with lower educational attainment associated with reduced rabies awareness, lower dog vaccination coverage, and weaker engagement with PEP. Evidence from Côte d’Ivoire and Tanzania similarly indicates that misconceptions and limited health literacy, rather than shortages of clinics or vaccines, are key barriers to the timely initiation of PEP and adherence [28]. These findings reinforce that service availability alone is insufficient; effective rabies control requires communication strategies tailored to community knowledge levels and risk perceptions [4,37]. In this study, the Surveillance Gap Index (SGI) and decision tree analyses further demonstrated that communities with lower awareness, education, and access to reliable information exhibited poorer surveillance performance and delayed responses. Collectively, these results highlight that strengthening rabies surveillance extends beyond technical infrastructure to include community empowerment and participatory, One Health-aligned approaches that integrate behavioral insight with human and veterinary health systems. At the local level, One Health coordination occurs through collaboration between primary healthcare clinics, state veterinary services, and community leaders, particularly during vaccination campaigns and outbreak response. Community health workers and animal health technicians play a critical bridging role in reporting bites, mobilizing households, and reinforcing prevention messages.

The COVID-19 pandemic caused substantial structural disruption within an already constrained health system. Although 45% of respondents reported interruptions to rabies-related services, the comparatively high post-pandemic dog vaccination coverage (85.7%) suggests a degree of community resilience and adaptive response. Similar patterns have been documented elsewhere, where pandemic-era adaptations, such as informal community support mechanisms and more flexible vaccination campaigns and delivery, helped sustain or restore preventive services [38,39]. These observations suggest that resilience extends beyond recovery to pre-pandemic functioning, encompassing the capacity of systems and communities to adapt through cross-sector collaboration and local participation [40,41,42,43]. Taken together, the findings support a shift in rabies control from predominantly reactive, clinic-based approaches toward proactive, integrated One Health strategies. Such approaches must account for the interconnected influence of social, behavioral, and structural determinants. Strengthening rabies surveillance and prevention, therefore, requires not only improved access to services but also knowledge, trust, and support systems that enable communities to engage effectively with available interventions. Ultimately, a resilient One Health system depends on both institutional preparedness and empowered communities.

### Study Limitations

This study has several limitations. As a pilot study with 109 participants, the findings are exploratory and may not be generalizable to all rural Eastern Cape communities. Machine learning analyses were applied to a small dataset; therefore, the results should be interpreted as hypothesis-generating rather than predictive or confirmatory. The cross-sectional design precludes causal inference, and self-reported behavioral data are subject to recall bias. Environmental risk factors were assessed indirectly through settlement and access characteristics rather than direct ecological measures. Finally, precise community-level dog vaccination coverage was unavailable, limiting the ability to quantify structural gaps in rabies control fully.

## 5. Conclusions

The interaction of social and structural determinants shapes rabies prevention and surveillance in rural South Africa. Education and health literacy underpin preventive behaviors, while reliable, accessible services are necessary to translate knowledge into timely action. The post-pandemic recovery observed in this study suggests that health systems anchored in community participation and adaptive learning can enhance vaccination uptake and surveillance responsiveness, even following periods of disruption. Achieving sustainable rabies elimination will therefore require more than expanding service provision; it necessitates embedding social insight, behavioral science, and system resilience within One Health policy and practice. By recognizing the interdependence of human behavior, animal health management, and health system capacity, South Africa can progress toward more equitable, integrated, and sustainable rabies control, with relevance for the prevention of other zoonotic diseases.

## Figures and Tables

**Table 1:** Socio-demographic and awareness characteristics of participants (N = 109).

Variable/Indicator	Category/Type	Findings or Proportion (%)
Age	Continuous	Mean ≈ 30.5 years (range 18–65)
Gender	Binary	Female 52%, Male 48%
Education level	Categorical	Secondary 55%, Primary 25%, Tertiary 20%
Occupation	Categorical	Unemployed 40%, Informal 35%, Formal 25%
Distance to health facility	Ordinal	>5 km 68%
Heard of rabies	Awareness indicator	88%
Correctly identified the cause (animal bite)	Awareness indicator	70%
Knew dogs could transmit rabies	Awareness indicator	95%
Attended a rabies awareness event	Awareness behavior	35%
Aware of free vaccination campaigns	Awareness behavior	42%
Household dog-bite experience	Exposure behavior	51%
Sought clinical care after a bite	Preventive practice	65% of bitten households
Household pets vaccinated	Preventive practice	76%
Vaccination by a professional veterinarian	Preventive practice	60%
No action taken after bite (“did nothing”)	Behavioral risk	15%
COVID-19 affected access to services	System impact	45%
Most reported disruptions	Qualitative summary	Travel restrictions (60%), fear of infection (25%), clinic closure (15%)

**Table 2:** Predictors of dog-bite exposure among participants.

Variable	Adjusted OR	95% CI	*p*-Value
Male gender	4.33	1.67–11.26	0.003 *
Education (level)	1.12	0.63–1.98	0.710
Distance > 5 km	0.12	0.02–0.90	0.039 *
COVID-19 impact (Yes)	1.63	0.62–4.33	0.323
Heard of rabies (Yes)	4.54	0.70–29.67	0.114

* means significant *p*-value.

## Data Availability

Data are available from the corresponding author upon request.
